# From Hope to Infection: Tubo‐Ovarian Abscess After Frozen Embryo Transfer—A Case Report and Review of the Literature

**DOI:** 10.1155/crog/5330571

**Published:** 2026-06-22

**Authors:** Ali El Safadi, Lara Nahouli, Macram Wakim, Antoine Hannoun

**Affiliations:** ^1^ Department of Obstetrics and Gynecology, American University of Beirut Medical Center, Beirut, Lebanon, aubmc.org.lb

**Keywords:** assisted reproductive technology, case report, endometriosis, frozen embryo transfer, tubo-ovarian abscess

## Abstract

Tubo‐ovarian abscess is a rare but serious complication of assisted reproductive technologies, typically following oocyte retrieval. We report a case of a 41‐year‐old woman with endometriosis who developed a tubo‐ovarian abscess 2 weeks after a hormonally prepared frozen embryo transfer. The procedure was uneventful and performed under sterile conditions with antibiotic prophylaxis. She later presented with pelvic pain and fever; imaging confirmed a right‐sided multiloculated adnexal abscess. Given the patient′s stable condition and abscess location, conservative management was chosen. Broad‐spectrum intravenous and oral antibiotics led to complete resolution without the need for drainage or surgery. This case highlights that tubo‐ovarian abscesses can occur even in low‐risk frozen embryo transfer cycles and in the absence of procedural complications. A review of the literature suggests that underlying pelvic pathology, including endometriosis, may predispose patients to infection. Considerate post‐transfer monitoring and individualized management strategies are essential to ensure favorable outcomes.

## 1. Introduction

Pelvic infections after assisted reproductive technologies (ARTs) are rare but potentially serious complications. Severe infections such as tubo‐ovarian abscess (TOA) occur in less than 1% of in vitro fertilization (IVF) cycles [1], and when they do, they are usually linked to oocyte retrieval rather than embryo transfer. Nonetheless, even a seemingly simple frozen embryo transfer (FET) can sometimes carry unexpected risks. Although cases of pelvic inflammatory disease (PID) or TOA following FET are exceptionally uncommon, physicians must remain vigilant. These rare occurrences typically involve patients with predisposing factors such as endometriosis or hydrosalpinx. We report a case of TOA following a hormonally stimulated FET cycle, and we discuss the clinical course and relevant literature.

## 2. Case Presentation

### 2.1. Patient′s History

A 41‐year‐old G1P1A0 presented for a FET. The patient had a history of infertility secondary to endometriosis and had previously undergone laparoscopic left ovarian cystectomy for a left ovarian endometrioma that was causing severe dysmenorrhea. Intraoperative findings at that time were consistent with Stage III endometriosis according to the revised American Society for Reproductive Medicine classification. Complete excision of the cyst wall was performed.

At the age of 35, she underwent egg retrieval and fresh embryo transfer at our institution. Eight blastocysts developed on Day 5, out of which two embryos were transferred and six blastocysts were frozen in two straws. The transfer was uncomplicated and resulted in a successful pregnancy. She delivered at 38 weeks and 3 days via urgent cesarean section for a nonreassuring fetal heart tracing.

### 2.2. Current ART Cycle and Embryo Transfer

Prior to the current FET cycle, 6 years later, an ultrasound demonstrated a small recurrent 1.5‐cm left ovarian endometrioma without other signs of active endometriosis. A hormonally stimulated FET cycle was initiated. She was premedicated with oral estradiol valerate 6 mg and folic acid. Five days prior to transfer, dydrogesterone and vaginal micronized progesterone gel 8% were initiated. In addition, prophylactic doxycycline 100 mg twice daily was started 5 days before embryo transfer as part of our institutional protocol to reduce the risk of ascending pelvic infection. Routine pretransfer infectious screening for *Chlamydia trachomatis* and *Neisseria gonorrhoeae* was not performed, as it is not part of our institutional protocol in patients without a history of PID or high‐risk sexual behavior.

The embryo transfer was performed under sterile conditions. The cervix was prepped with normal saline, and all cervical mucus was removed, and the cervix appeared normal. A soft catheter was initially introduced by the primary physician to ensure patency into the endometrial cavity. Subsequently, three blastocysts were loaded into another soft catheter and transferred smoothly under abdominal ultrasound guidance. Given the patient′s age and reduced implantation potential associated with advanced maternal age, the decision to transfer three not genetically tested blastocysts was individualized following counseling regarding the increased risk of multiple pregnancies.

Following the procedure, the patient was discharged on estradiol valerate 6 mg, dydrogesterone, vaginal progesterone gel 8%, and low‐dose acetylsalicylic acid. Ten days later, serum beta‐human chorionic gonadotropin (*β*‐hCG) came back negative, indicating implantation failure.

## 3. Clinical Presentation

Two weeks post‐transfer, the patient presented to the clinic with sharp right‐sided pelvic pain, rated 9/10 in severity, partially relieved by analgesics. She also reported a fever up to 38.9°C. On abdominal examination, right lower quadrant tenderness was noted, without rebound tenderness. Speculum examination revealed yellowish vaginal discharge. Bimanual examination demonstrated marked right adnexal tenderness without cervical motion tenderness.

### 3.1. Diagnostic Assessment

A transvaginal ultrasound revealed a 6 × 5 cm complex right adnexal mass. The patient was referred to the emergency department. In the emergency room, the patient′s vital signs showed she was afebrile (36.8°C) but tachycardic (heart rate 112 bpm). Laboratory investigations revealed a white blood cell count of 9200/*μ*L (82% neutrophils) and thrombocytosis (platelet count 501,000/*μ*L). C‐reactive protein (CRP) was elevated at 131 mg/L (normal less than 5). A repeat serum *β*‐hCG remained negative, confirming she was not pregnant. Contrast‐enhanced computed tomography (CT) of the abdomen and pelvis demonstrated a 6.2 × 5.4 × 5.1 cm thick‐walled, multiloculated collection in the right adnexa with peripheral enhancement and significant fat stranding. The mass displaced the uterus to the left and was adherent to the tip of the appendix on imaging. These findings were consistent with a TOA involving the right fallopian tube and ovary, as shown in Figures 1 and 2. Differential diagnoses included appendiceal abscess and complicated ovarian cyst; however, imaging characteristics and clinical findings were most consistent with TOA. A vaginal swab for sexually transmitted infection screening was offered, but the patient declined.

**Figure 1:**
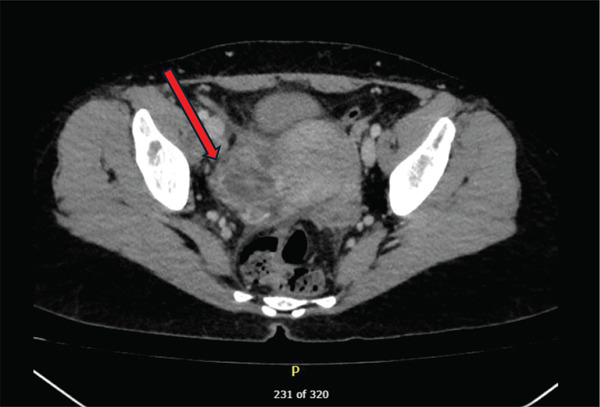
Axial CT image showing a multiloculated right tubo‐ovarian abscess (arrowed) with surrounding inflammatory changes.

**Figure 2:**
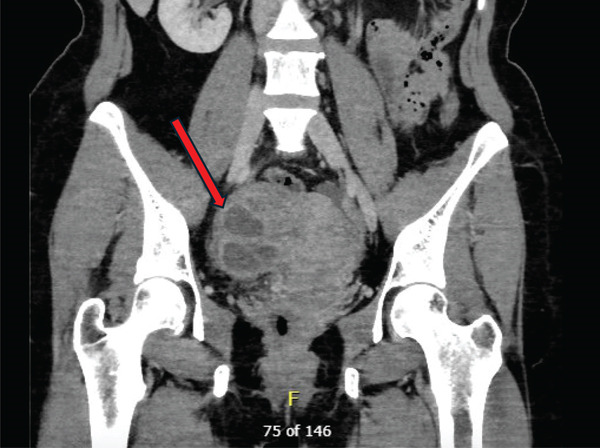
Coronal CT image demonstrating displacement of the uterus and adjacent inflammatory changes associated with the abscess (arrowed).

### 3.2. Therapeutic Intervention

Broad‐spectrum antimicrobial therapy was initiated in consultation with the infectious diseases service. The patient was started on intravenous piperacillin–tazobactam 4.5 g every 6 h along with continuing doxycycline 100 mg twice daily. Interventional radiology was consulted to evaluate the possibility of percutaneous drainage of the abscess. However, drainage was deemed high‐risk due to the abscess′s proximity to bowel loops and its adherence to the appendix. Surgical intervention was also deferred as the patient was clinically stable and there were no signs of rupture.

### 3.3. Follow‐Up and Outcomes

The patient showed clear improvement within 48 h of antibiotic therapy. Her fever did not recur, and her pelvic pain significantly decreased. Inflammatory markers began to trend down (CRP decreased to 78.7 mg/L by the second hospital day). Repeat pelvic ultrasound demonstrated that the abscess was not enlarging and had begun to decrease in size under conservative management. Given her stable clinical course, the decision was made to continue nonsurgical management. After 3 days of inpatient treatment, the patient was discharged home on a regimen of oral antibiotics: amoxicillin–clavulanate 1 g twice daily, ciprofloxacin 500 mg twice daily, and doxycycline 100 mg twice daily, to cover a broad spectrum of possible pelvic pathogens. The total duration of antibiotic therapy was 14 days. The patient tolerated the antibiotic therapy well without reported adverse drug reactions.

At outpatient follow‐up 10 days after hospital discharge, the patient reported complete resolution of pelvic pain and had no fever. Transvaginal ultrasound at that time showed the right adnexal abscess had reduced in size to approximately 4 cm in diameter. Fourteen days after completing antibiotics, her CRP had normalized. By 6 weeks after the initial presentation, imaging confirmed complete resolution of the abscess. She was advised to defer further embryo transfer for at least 3–6 months and to undergo repeat imaging and STI testing before attempting another transfer.

### 3.4. Timeline

At age 33, the patient underwent laparoscopic left ovarian cystectomy for endometriosis. At age 35, she underwent IVF with fresh embryo transfer, resulting in a live birth. At age 41, she underwent FET. Ten days after transfer, *β*‐hCG was negative. Fourteen days after transfer, she presented with pelvic pain and fever and was diagnosed with TOA. She received intravenous antibiotics followed by 14 days of oral therapy. Six weeks after diagnosis, imaging demonstrated complete resolution.

## 4. Patient Perspective

The patient described the complication as unexpected and distressing, particularly following what she perceived as a routine FET. She expressed concern regarding the possible impact on her future fertility but felt reassured by the favorable response to conservative treatment and the absence of need for surgical intervention.

## 5. Discussion

TOA is an uncommon but significant complication following embryo transfer. Within the broader context of assisted reproduction, most pelvic abscesses have been reported after oocyte retrieval, a procedure involving transvaginal instrumentation. Severe infection after oocyte aspiration remains rare, occurring in well under 1% of cases [1]. Routine measures such as prophylactic antibiotics and strict aseptic technique are generally effective at minimizing risk [2]. In our case, despite the patient receiving prophylactic doxycycline and undergoing a smooth, sterile embryo transfer, she still developed a TOA. Current ESHRE guidelines recommend elective single embryo transfer as the standard approach to reduce multiple pregnancy risk; however, embryo number may be individualized in selected patients with lower predicted implantation potential after appropriate counseling [3]. This serves as a reminder that, although rare, serious infections can arise even when protocols are meticulously followed. Sauer and Paulson first documented a pelvic abscess after embryo transfer in 1992 [4]. Notably, their patient was an agonadal embryo recipient who had not undergone oocyte retrieval, underscoring that the embryo transfer itself carries a theoretical risk. Similarly, Friedler et al. described a ruptured TOA following a frozen‐thawed embryo transfer, despite routine antibiotic use, suggesting that contamination can still occur during transfer [5].

A review of the literature highlights that many cases of TOA or severe pelvic infection associated with IVF involve identifiable risk factors. Patients with underlying pelvic pathology appear particularly vulnerable. Younis et al. reported late‐onset pelvic abscesses following IVF in women with severe endometriosis and endometriomas [6]. Endometriotic cysts may offer an ideal environment for secondary bacterial infection. In another report, a woman with known hydrosalpinx, likely a sequela of prior PID, developed a TOA after IVF; it was proposed that a dormant infection in the damaged fallopian tubes reactivated during hormonal stimulation [7]. In our patient, there was a history of endometriosis treated surgically, but no documented PID or hydrosalpinx. It is plausible that hormonal stimulation, in combination with transcervical instrumentation, facilitated ascending infection in a susceptible pelvic environment.

Unfortunately, cultures were not obtained in this case, limiting our ability to identify the pathogen. In similar cases, polymicrobial flora typical of PID is often isolated. Interestingly, a rare case reported by Asemota et al. involved *Actinomyces* species causing pelvic abscess after IVF, successfully managed with antibiotics and drainage without requiring removal of reproductive organs [8].

Management of TOA following IVF demands a personalized approach. Factors such as the patient′s clinical status, size of the abscess, and reproductive goals must guide decision‐making. For stable patients with moderate‐sized abscesses, conservative therapy with broad‐spectrum antibiotics may be sufficient, as demonstrated in our case. Early recognition and aggressive medical management can sometimes avoid the need for surgery.

There are similar examples in the literature. Yalcinkaya et al. described a 31‐year‐old IVF patient who developed bilateral ovarian abscesses shortly after oocyte retrieval. Broad‐spectrum antibiotics combined with transvaginal ultrasound–guided drainage allowed the pregnancy to continue, culminating in a term delivery [9]. Similarly, Sharpe et al. reported an ovarian abscess in an IVF twin pregnancy presenting later in gestation, which was managed conservatively until spontaneous vaginal delivery at term [10]. Our case and theirs highlight that nonsurgical management is feasible in select cases, even in early pregnancy or recent embryo transfer.

Nevertheless, many reported TOA cases ultimately required surgical intervention, often because of abscess size, failure to respond to antibiotics, or risk of rupture. When feasible, laparoscopy offers a minimally invasive option, particularly when preserving a pregnancy is a priority. Kim et al. reported a case where laparoscopic drainage of a TOA during early pregnancy allowed the patient to deliver at term [11]. However, there are sobering cases where emergency laparotomy was unavoidable. Friedler et al.′s 1996 case involved a ruptured TOA requiring immediate surgical management [5]. Biringer et al. described managing a severe pelvic abscess during a twin pregnancy via laparotomy, successfully delaying delivery [12]. Conversely, some infections, even when promptly recognized, led to pregnancy loss. Matsunaga et al. reported a TOA during an IVF pregnancy that, despite antibiotic therapy, resulted in preterm labor and neonatal loss at 22 weeks [13]. A separate case from Taiwan involved a ruptured TOA in the first trimester necessitating surgery, with loss of the pregnancy [14]. These outcomes underscore how timing, severity, and rapid intervention can dramatically affect prognosis. Table 1 provides a summary of documented TOA or PID cases after embryo transfer over recent decades. Of particular note, the largest series published to date by Fouks et al. showed that TOA can have lasting effects on ovarian reserve and future fertility. Many patients in that series had lower pregnancy rates after treatment, even if the infection itself was successfully managed [15]. Hormonal preparation for FET may contribute to infection through multiple mechanisms. Estrogen and progesterone exposure can alter cervical mucus composition and reduce its barrier function, thereby facilitating ascending microbial migration from the lower genital tract [16]. In addition, supraphysiologic hormonal environments may modulate local immune responses within the endometrium and fallopian tubes, impairing innate host defense mechanisms and increasing susceptibility to infection [17]. Patients with endometriosis may be particularly vulnerable due to distorted pelvic anatomy, altered peritoneal immune function, and the presence of endometriotic lesions that may serve as a nidus for bacterial colonization [6, 18]. Furthermore, transcervical embryo transfer may introduce vaginal or cervical flora into the upper genital tract despite adherence to sterile technique, particularly in the setting of repeated instrumentation [5, 8]. These combined factors likely contribute to the rare occurrence of TOA following FET.

**Table 1:** Reported cases of tubo‐ovarian abscess (TOA) or PID after embryo transfer.

Authors	Year	Risk factors	Clinical presentation	Onset time	Bacterial study	Time to intervention	Management	Outcome	Maternal and neonatal outcomes	Reference
Sauer and Paulson	1992	None reported (agonadal recipient)	Pelvic pain and abscess	Not specified	Not reported	Delayed	Antibiotics and surgery	Recovered	Not applicable	[4]
Friedler et al.	1996	Not specified	Ruptured TOA	Early post‐FET	Not reported	Emergency	Surgery	Recovered	Not applicable	[5]
Younis et al.	1997	Severe endometriosis and endometrioma	Pelvic abscess	Late onset	Not reported	Delayed	Surgical	Recovered	Not applicable	[6]
Matsunaga et al.	2003	IVF pregnancy	TOA during pregnancy	20 weeks of gestation	Not reported	Delayed	Antibiotics then surgery	Preterm delivery	Neonatal loss	[13]
Varras et al.	2003	Hydrosalpinx and prior PID	Ruptured TOA	Early post‐ET	Not reported	Emergency	Surgery (salpingo‐oophorectomy)	Recovered	Pregnancy not preserved	[7]
Sharpe et al.	2006	IVF twin pregnancy	Ovarian abscess and mild symptoms	Late onset	Not reported	Delayed	Conservative	Resolved	Term twin delivery	[10]
Biringer et al.	2009	IVF twin pregnancy	Severe pelvic abscess	Mid‐trimester	Not reported	Urgent	Laparotomy	Improved	Pregnancy prolonged	[12]
Yalcinkaya et al.	2011	Recent oocyte retrieval	Bilateral abscess	9 days post‐IVF	Not reported	Early	Antibiotics + drainage	Resolved	Term delivery	[9]
Kim et al.	2013	IVF pregnancy	TOA in early pregnancy	First trimester	Not reported	Early	Laparoscopy + antibiotics	Resolved	Term delivery	[11]
Asemota et al.	2013	None reported	Pelvic abscess	6 days post‐ET	Actinomyces	Early	Antibiotics + drainage	Resolved	Fertility preserved	[8]
Han et al.	2015	IVF pregnancy	Ruptured TOA	Early pregnancy	Not reported	Emergency	Laparotomy	Severe infection	Pregnancy loss	[14]
Fouks et al.	2019	Post‐oocyte retrieval	TOA cases (series)	Variable	Not specified	Variable	Mixed	High morbidity	Reduced fertility outcomes	[15]
El Safadi et al. (current case)	2026	Endometriosis and prior surgery	Fever, pelvic pain	14 days post‐FET	Not obtained	Immediate	IV then switched to oral antibiotics	Complete resolution	No pregnancy; full recovery	**—**

Our case adds to this body of literature as an example of a TOA following a frozen‐thaw embryo transfer in a patient with endometriosis. Uniquely, the infection occurred in the absence of a recent oocyte retrieval or an ongoing pregnancy, and it was successfully managed with medical therapy alone. It reinforces the need for fertility specialists to maintain a high index of suspicion when patients present with pelvic pain and fever after embryo transfer. If a TOA is confirmed, a multidisciplinary approach involving fertility specialists, radiologists, and infectious disease experts is essential. Early imaging and prompt initiation of broad‐spectrum antibiotics are critical to prevent progression to rupture and preserve reproductive potential.

## 6. Limitations

This report is limited by the absence of microbiological confirmation and the patient′s decision to decline STI testing at presentation. In a similar future case, we would prioritize obtaining cultures, where feasible, and standardized STI testing at presentation to better guide targeted therapy. As a single case report, generalizability is limited. Nevertheless, it contributes to the limited literature describing TOA following FET.

## 7. Conclusion

TOA following FET is a rare but serious complication. Our case highlights that even with meticulous technique and prophylaxis, infections can still arise, particularly in patients with underlying risk factors such as endometriosis. Prompt diagnosis and initiation of broad‐spectrum antibiotics are key to successful conservative management. Awareness of this possibility ensures timely intervention, optimizing outcomes and preserving reproductive potential. Ongoing reporting of such cases will continue to inform best practices for the prevention and management of this uncommon complication.

## Funding

No funding was received for this manuscript.

## Ethics Statement

Written informed consent was obtained from the patient for the publication of this case report and accompanying images. Institutional review board approval was not required for anonymized single case reports according to institutional policy.

## Conflicts of Interest

The authors declare no conflicts of interest.

## Data Availability

The data that support the findings of this study are available upon request from the corresponding author. The data are not publicly available due to privacy or ethical restrictions.
